# An Inductively Powered Implantable System to Study the Gastrointestinal Electrophysiology in Freely Behaving Rodents

**DOI:** 10.3390/bioengineering9100530

**Published:** 2022-10-06

**Authors:** Dylan T. Berry, Joanne Choi, Calla A. Dexheimer, Morgan A. Verhaalen, Amir Javan-Khoshkholgh

**Affiliations:** Bioinstrumentation and Medical Diagnostic Systems (BioMDS) Laboratory at the Department of Materials Science and Biomedical Engineering, The University of Wisconsin—Eau Claire, Eau Claire, WI 54702, USA

**Keywords:** adaptive wireless power transfer, bidirectional data communication, gastric bioelectrical activity, gastric recording and stimulation, near-field communication, specific absorption rate

## Abstract

Chronic studies in the fasting and fed states of conscious subjects are fundamental for understanding the pathophysiological significance of functional gastrointestinal (GI) disorders and motility dysfunctions. To study the electrophysiology of the GI tract in the long term, the development of gastric implants is essential. This paper presents the development of an implantable system capable of monitoring the bioelectrical activity of the gastric system and modulating the activity in freely behaving rodents. The system consists of a miniature-sized implantable unit (IU), a stationary unit (SU) that communicates with the IU over a 2.4 GHz far-field radio frequency (RF) bidirectional link, and a charging unit (CU) that establishes an inductive 13.56 MHz near-field communication (NFC) with the IU, implementing an adaptive wireless power transfer (WPT). The CU can generate an adjustable power between +20 dBm and +30 dBm, and, in the presence of body movements and stomach motility, can deliver a constant rectified voltage to the IU. The live subject’s exposure to the electromagnetic WPT in the developed system complies with the RF energy absorption restrictions for health and safety concerns. The system can be utilized to investigate the relationship between functional GI disorders and dysrhythmias in the gastric bioelectrical activity and study the potential of electroceutical therapies for motility dysfunctions in clinical settings.

## 1. Introduction

Functional gastrointestinal (GI) tract diseases, particularly gastroparesis and functional dyspepsia, are neuromuscular motility disorders in the gastric system that involve both motor and sensory dysfunctions and are increasingly recognized as a cause of chronic abdominal symptoms [1,2,3]. Functional dyspepsia is a highly prevalent and chronic GI disorder, impacting 15.8%–31.9% of the population of the United States [4]. It can be diagnosed using the symptom-based Rome IV criteria and is characterized by symptoms of postprandial fullness, early satiation, epigastric pain, and/or epigastric burning [5,6]. In addition, functional dyspepsia is associated with depression and generalized anxiety disorder [7,8]. Furthermore, gastroparesis is a debilitating condition characterized by delayed gastric emptying in the absence of mechanical obstruction, with symptoms including postprandial fullness, nausea, vomiting, and bloating [1,9,10,11]. These disorders may be part of a spectrum of gastrointestinal neuromuscular disorders [12]. A measurable delay in gastric emptying is associated with neuropathic disorders and, in particular, diabetes; it affects about 40% of patients with type 1 diabetes and up to 30% of patients with type 2 diabetes [13,14].

An underlying bioelectrical form of activity, known as slow waves (SWs), initiates and coordinates the motility of the GI system [15]. In acute studies on humans, it has been demonstrated that functional gastric disorders cause aberrant SW patterns and detectable dysrhythmias in the electrophysiological activity of the stomach [16,17,18]. In the absence of effective medications, electroceutical therapies and electrical stimulation pulses, when applied directly to the GI tract, have demonstrated merits in improving gastric motility, relieving functional GI disorders, and accelerating gastric emptying. While high-energy, low-frequency pulses with pulse-widths in the order of hundreds of milliseconds have shown efficiency in modulating and pacing the activity of the SWs, low-energy high-frequency pulses with pulse-widths of a few hundreds of microseconds can be helpful in relieving nausea and vomiting [19,20]. However, the pathophysiological roles of abnormalities in functional GI disorders and neuromuscular dysfunctions, as well as the therapeutic potential of electroceutical methods, remain incompletely investigated. Lack of technology, invasive and wired systems with the risk of dislodgement and infection, the absence of devices for simultaneously recording and stimulating the SWs activity, and a lack of implantable medical devices (IMDs) featuring a wireless power transfer (WPT) link for conducting research on the gastric system have been the major challenges in terms of studying the mechanism of GI dysrhythmias in the long term [20].

Regarding the short range-to-size ratio for gastric IMDs, resonant inductive WPT is considered the optimum technique and is widely used to supply power for gastric implants, wirelessly. However, since the inductive WPT propagates electromagnetic (EM) waves and exposes live tissues to a radio frequency (RF) EM field, the adverse health effects of exposure to EM fields are of major concern. To this end, a specific absorption rate (SAR) that is equal to the rate of absorbed RF energy is of paramount importance and is expressed as the time rate of temperature change in °C/s times the specific heat capacity expressed in J/(kg °C). IMDs need to follow the regulations set by the Federal Communications Commission (FCC) and according to the IEEE standard for safety levels with respect to human exposure to electric, magnetic, and electromagnetic fields; therefore, the SAR of partial body exposure is limited to 2 W/kg over 10 g of tissue [21]. In addition, in terms of medical implants developed for gastric systems, significant body movements and the stomach motility of the subject cause misalignment and/or changes of distance between the transmitter and receiver coils on the inductive link, resulting in a decreased or excessive amount of power received by the implant. As a result, adaptive WPT is an essential feature that must be developed in gastric IMDs.

To address the power supply concerns regarding medical implants and overcome the corresponding limitation, several systems for GI tract or neural studies with wireless power transfer links have been developed to either power the IMDs directly, or recharge their batteries through resonant inductive links [22,23,24,25,26,27,28,29]. However, these systems support either the recording or stimulation functions [22,23,24], both of which are necessary for bidirectional interfacing, or else the wireless power is transmitted in an open-loop fashion [25,26,27,28,29]. To comply with the SAR requirements, a closed-loop system needs to control the WPT link instantaneously. In addition, the system needs to integrate both the recording and stimulation functions with bidirectional data communication. To this end, we developed a miniature implantable system for simultaneously recording and modulating the SWs in freely behaving rodents. The featured system is supplied with a radio frequency identification (RFID)-based closed-loop WPT, which acts within the safety limits of the SAR and delivers a constant level of power to the gastric implant. 

## 2. Materials and Methods

**Figure 1:**
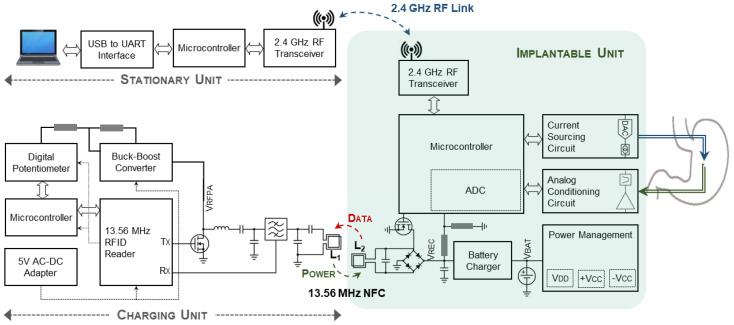
A detailed block diagram of the developed system to study gastric electrophysiology, consisting of an implantable unit (IU), the charging unit (CU), and the stationary unit (SU) connected to a computer.

The detailed block diagram of the system used to study the electrophysiology of the GI tract is shown in Figure 1. It consists of a miniature implantable unit (IU), a charging unit (CU), and a stationary unit (SU) connected to a computer. Two communication links have been developed for the system. A 2.4 GHz transceiver establishes bidirectional RF data communication between the IU and the SU. The IU transmits the conditioned and digitized SWs to the SU, while on the reverse link, the user at the base station sends the stimulation configurations. Furthermore, an NFC establishes an inductive wireless power and data transfer link between the CU and the IU at a carrier frequency of 13.56 MHz, such that it delivers constant power to the IU through an RFID-based closed-loop system. To this end, the rectified voltage (V_REC_) at the IU is compared to a reference voltage (V_REF_) that is programmed at the CU to adjust the transmitting power, by adjusting the power supply of the RF power amplifier instantaneously. Both the chosen frequencies of 13.56 MHz used for NFC communication and 2.4 GHz for RF communication are in those parts of the spectrum that are internationally reserved for the use of industrial, scientific, and medical (ISM) applications.

### 2.1. Implantable Unit—Recording

**Figure 2:**
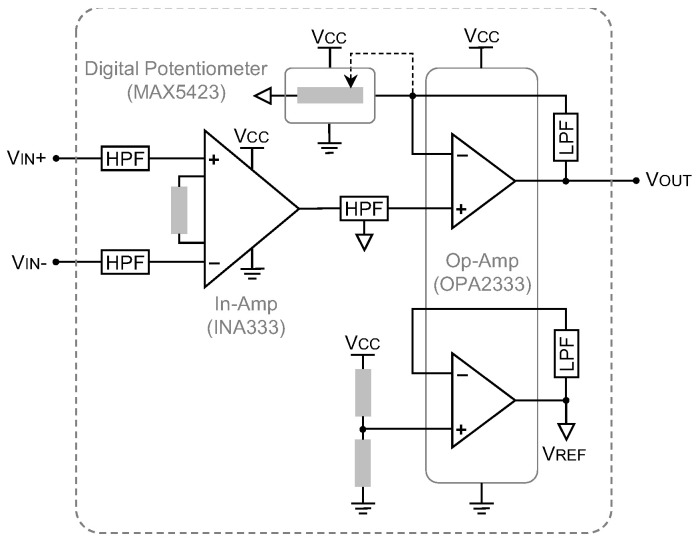
The schematic of the analog conditioning circuit for recording gastric bioelectrical activity is shown. It amplifies the SWs in two stages, with a wirelessly programmable gain, and band-pass filters the signals at 16 mHz–1.6 Hz. The analog conditioning circuit can sense SWs with amplitudes as low as 100 µV_PP_.

To record the gastric electrophysiology, a two-stage analog conditioning circuit acquires the SWs and provides the amplified and filtered waves at the output to an analog-to-digital converter (ADC), embedded in a microcontroller (MSP430, Texas Instruments). In the first stage, the SWs are pre-amplified through an instrumentation amplifier (INA333, Texas Instruments) with a constant gain of 10 and are high-pass-filtered at a cutoff frequency of 16 mHz. Afterward, an active low-pass filter, developed based on an operational amplifier (OPA2330, Texas Instruments), removes the noise and artifacts seen with frequencies higher than 1.6 Hz. The gain of the active filter can be instantaneously programmed using a 100 kΩ 256-tap programmable digital potentiometer (MAX5423, Maxim Integrated) on the feedback loop of the amplifier. As a result, based on the peak-to-peak voltage of the acquiring SWs, the total gain of the recording circuit can be programmed to between 110 and 25500, to reach an optimum amplitude at the input of the ADC. It is then sampled by the ADC at a sampling rate of 20 samples per second and a resolution of 12 bits. Figure 2 shows the schematic of the analog conditioning circuit.

### 2.2. Implantable Unit—Stimulation

In general, to modulate the electrophysiological activity of the GI tract, both voltage-controlled and current-controlled stimulation pulses can be applied. However, due to the heterogeneous bio-impedance of the gastric tract, as well as the inconstant impedance of the electrode-tissue interface over time, voltage-controlled pulses may not be able to generate a uniform voltage distribution across the stimulation electrodes in the stomach. Delivering a constant charge per pulse in current-controlled stimulations minimizes the issue; besides, it helps to minimize the changes in the long-term therapeutic efficacy of stimulation [30,31]. As a result, we developed a stimulation circuit based on generating current-controlled pulses. It delivers monophasic and biphasic electrical pulses to the GI tract. Through the IU’s microcontroller, square-wave pulses with amplitudes of up to ± 10 mA, a minimum pulse width of 50 μs, and a maximum frequency of 10 kHz are first programmed and then generated through a digital-to-analog converter (DAC8771, Texas Instruments) with an embedded buffered current source. In addition, the stimulation pulses can be configured to be symmetric or asymmetric.

### 2.3. Implantable Unit—Communication

**Figure 3:**
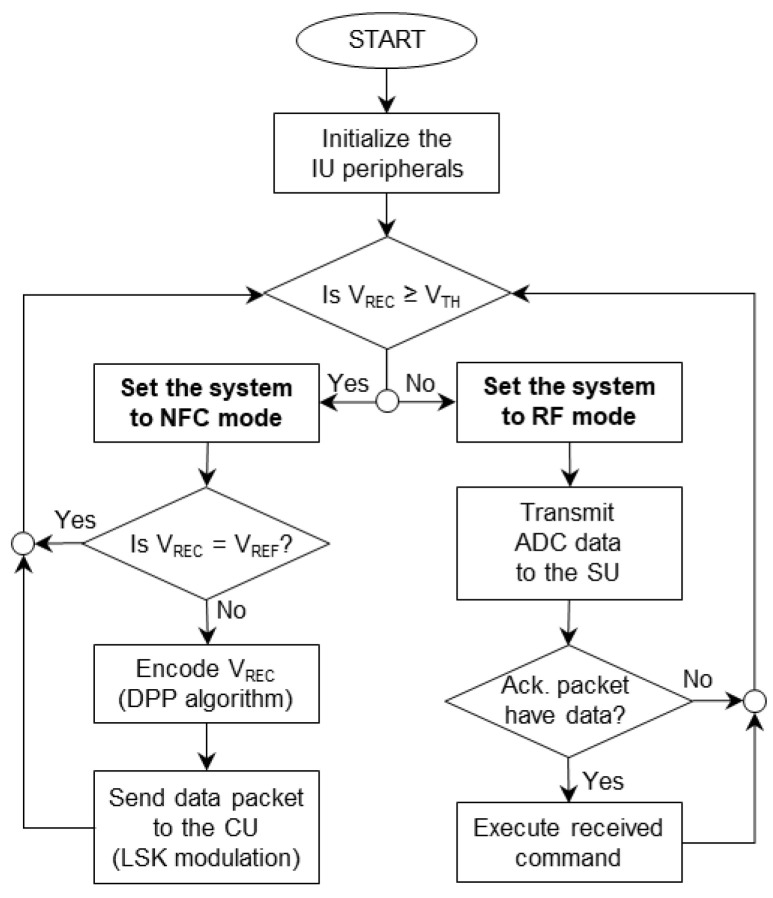
The flowchart of the sequence of operation of the IU.

The IU is capable of operating in two distinct modes of RF and NFC and can toggle between them. While the RF mode is assigned to study the electrophysiology of the GI tract in freely behaving rodents, i.e., the recording and stimulation of the SWs, the IU’s battery is recharged in the NFC mode. The flowchart of the operation of the IU is shown in Figure 3. By default, the IU operates in RF mode. However, as soon as it detects a V_REC_ that is greater than the programmable threshold of V_TH,_ with an initial value of 1 V, it switches to the NFC mode. This is one of the unique features of the system and eliminates the necessity of using a magnetic switch on the IU.

In RF mode, a low-power transceiver (nRF24L01+, Nordic Semiconductor) establishes a bidirectional data communication at 2.4 GHz between the IU and the SU. On the uplink, the digitized SWs data are transmitted to the SU in real time, while on the downlink, the stimulation configurations, as well as the instruction to modify the gain of the analog conditioning circuit, are sent by the stationary user. The carrier frequency of the RF link is programmable in the range of 2.4 GHz to 2.5 GHz in steps of 2 MHz and, as a result, a minimum of 50 independent IUs can be paired with their corresponding SUs, as seen in clinical studies. 

Furthermore, the under-the-cage CU establishes an inductive 13.56 MHz NFC with the IU and implements a wireless power and data transfer through an RFID-based closed-loop control system. In the NFC mode and on the reverse path of the WPT, the IU takes samples of the V_REC_, then encodes and sends them on to the CU. Since there is a single path for data transfer in the NFC mode, we developed a self-clocking algorithm of differential pulse position (DPP) based on the Manchester coding. According to the IEEE 802.3 convention, logic “0” and logic “1” are represented by transitions from “high” to “low” and from “low” to “high”, respectively, both with a 50% duty cycle. To optimize the WPT efficiency, we decreased the duty cycle of wireless data transfer to 6.25%, i.e., the IU interrupts the WPT for only one-eighth of the time needed to send each bit of data. As a result, for a data rate of 125 kb/s, we reduced “high” pulses from 4 μs to 0.5 μs. The DPP encoding algorithm is discussed by the authors of [32]. Each NFC data packet consists of an 8-bit start-of-frame (SOF) header and a 12-bit V_REC_ sample. Based on the 8 μs of time needed to send each bit, it takes only 0.16 ms for the IU to send the data packet to the CU.

One of the most effective power-saving and passive methods to modulate the data at the IU for low data transfer rates is load-shift keying (LSK) [33,34,35,36]. This can be implemented by switching the IU’s load on and off through a MOSFET transistor (Si2338, Vishay Intertechnology). For sending the “high” pulses of the data packet, the switch shorts the secondary coil (L_2_) to the ground and modifies the load resistance at the IU. The change of impedance at L_2_ can be seen in the primary coil (L_1_) with changes in the reflected impedance, which, in turn, changes the voltage level of the RF power signal at the CU. Therefore, the data packets can be detected using an envelope detector. The details of the LSK modulation are explained in a previous study [32]. 

**Figure 4:**
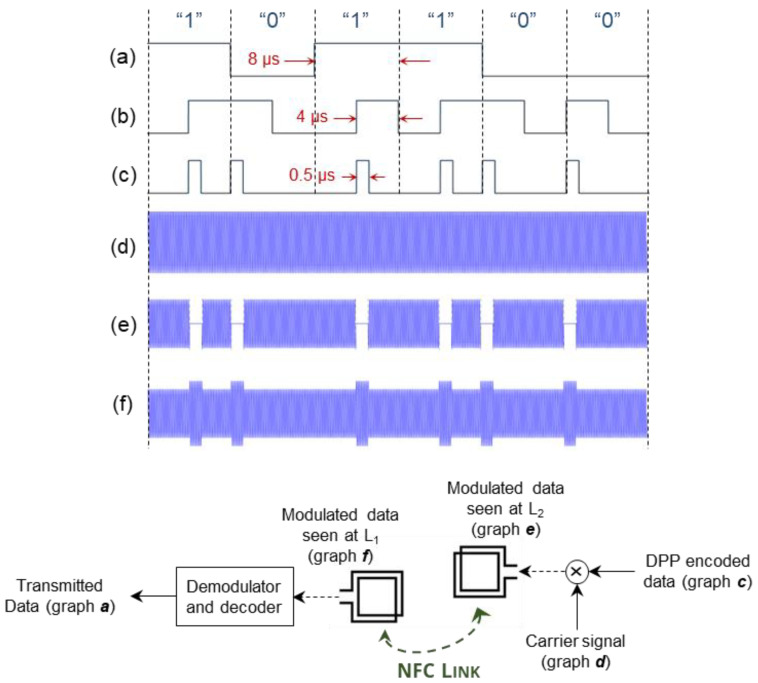
The schematic of the DPP data encoding and the LSK data modulation for the NFC mode of operation: (**a**) an arbitrary sequence of digital logic values of “1”s and “0”s, (**b**) IEEE 802.3 standard Manchester encoding with 50% duty cycle, (**c**) developed DPP encoding with only 0.5 µs high-pulse width. This is the encoded data to be sent over the inductive link. (**d**) Carrier signal at the frequency of 13.56 MHz, (**e**) the encoded data modulated over the carrier signal at the secondary coil, and (**f**) the signal that is detected by the primary coil, which is demodulated and then decoded at the CU.

Figure 4 graphically presents the DPP encoding and the LSK modulation, applied to an arbitrary 6-bit sequence of data at the IU, along with their demodulation and decoding at the CU. First, the IEEE 802.3 standard Manchester encoding with a 50% duty cycle is applied to the original data; and then, the duty cycle is reduced to 6.25% (see graph c in Figure 4. In the next step, it is streamed through the back-telemetry circuit at the IU. For high pulses, the L_2_ is shorted to the circuit ground; therefore, the received power is zero in graph e. Graph f demonstrates the change of voltage at L_1_ as a result of the change in impedance on L_2_, which is demodulated by the envelope detector, embedded at the RFID, and decoded by a microcontroller (MSP432, Texas Instruments) at the CU.

### 2.4. Implantable Unit—Power Management

The 3.7 V battery voltage at the IU is regulated to 3.3 V through a low-dropout linear regulator (TPS735, Texas Instruments), which supplies the analog conditioning circuit, the microcontroller, and the RF transceiver. In addition, to support both the monophasic and biphasic types of stimulation for current pulses with amplitudes of up to ±10 mA, the battery supplies a DC–DC boost and inverting converter (LT3471, Linear Technology), which generates DC voltages of ± 13 V and supplies the digital-to-analog converter. In the RF mode of operation, the battery voltage is sampled by the ADC through a voltage divider, then, along with the SWs data, it is transmitted to the SU. 

In the NFC mode, the wireless power delivered to L_2_ is rectified through a Schottky diode array (BAS3007, Infineon). Afterward, the output of the bridge rectifier, V_REC_, recharges the IU’s battery through a lithium–polymer charge management controller (MCP73831, Microchip).

### 2.5. Charging Unit

A class-E RF power amplifier at the CU generates adjustable power in the range of +20 dBm (100 mW) to +30 dBm (1000 mW) and transmits it to the IU through the inductive link. The design is based on an N-channel power MOSFET transistor (IRFR110, Vishay Intertechnology). In addition, L_1_ is designed as the planar type, with a narrow-band capacitive network to match the coil to 50 Ω. The RF power amplifier is driven through the RF output of a 13.56 MHz RFID (TRF7970, Texas Instruments). The RFID receives the data sent by the IU through a capacitive voltage divider at the output of the RF power amplifier and demodulates them through an embedded envelope detector. A DC-DC buck-boost converter (LTC3111, Linear Technology) supplies the RF power amplifier directly. By increasing/decreasing the resistance of a 200 kΩ 256-tap programmable digital potentiometer (MAX5424, Maxim Integrated) on the feedback loop of the DC-DC converter, the output power of the CU is decreased/increased accordingly. 

**Figure 5:**
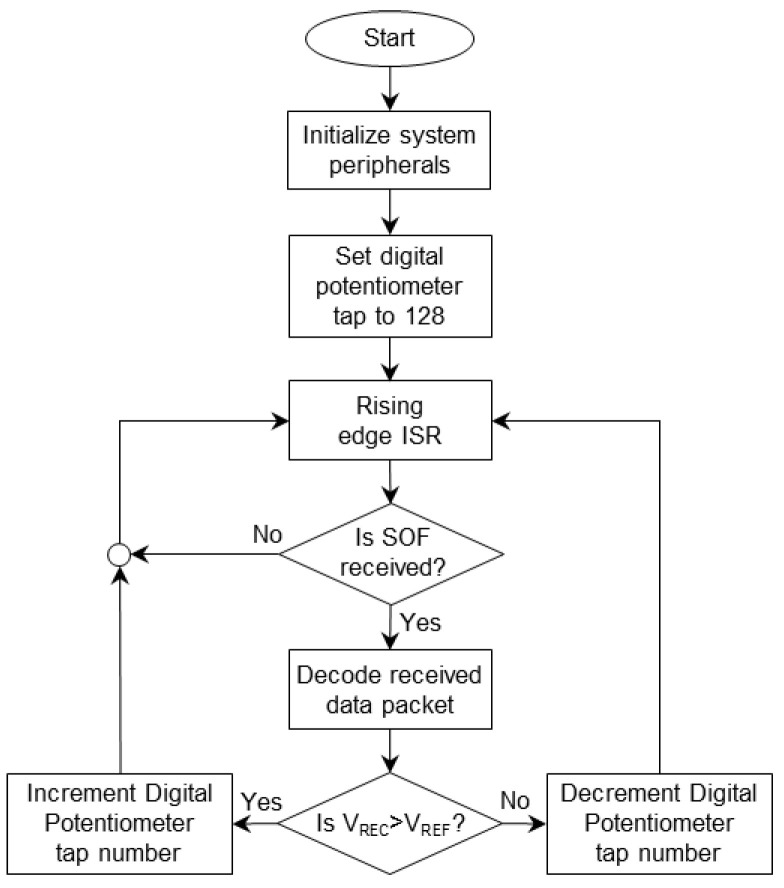
The flowchart of the adaptive closed-loop WPT function at the CU.

Upon detection of the SOF by the CU, the microcontroller decodes the received data, i.e., the instantaneous value of the V_REC_, and compares it to the V_REF_. For a V_REC_ greater than the V_REF_, the CU needs to decrease the transmitting power, such that the V_REC_ reduces down to the V_REF_. As a result, the CU increases the resistance of the digital potentiometer. Otherwise, in the case of a V_REC_ that is less than the V_REF_, the value of the digital potentiometer is decreased to increase the supply voltage of the RF power amplifier. Figure 5 shows the flowchart of the operation of the closed-loop WPT at the CU. In addition, Table 1 presents the lookup table for the incremental or decremental steps on the tap number of the potentiometer at the CU.

### 2.6. Stationary Unit

The stationary user communicates with the IU through the SU. It receives the data transmitted by the IU through the same 2.4 GHz RF transceiver; afterward, a microcontroller (MSP430, Texas Instruments) sends the data to the computer through a USB-to-UART interface (FT232R, FTDI) for monitoring and storing the acquired SWs and for further analysis. Besides, the user’s stimulation instructions, as well as the command to change the gain of the analog conditioning circuit, are transmitted to the IU through the SU. The 2.4 GHz RF link supports a range of 20 m between the IU and the SU in an open space.

### 2.7. Graphical User Interface

**Figure 6:**
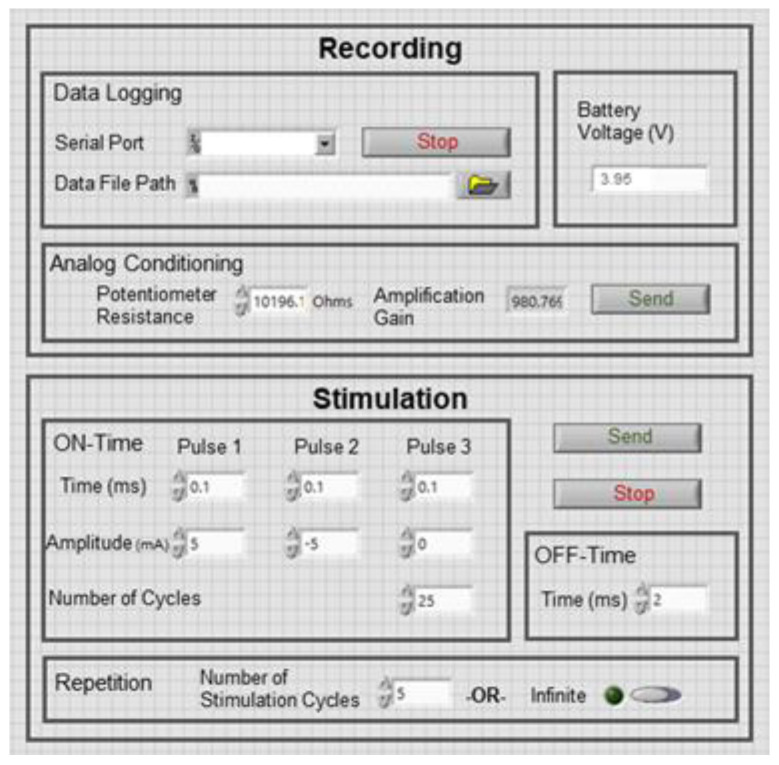
The programming panel of the graphical user interface developed for the IU-SU wireless bidirectional communication.

A graphical user interface (GUI) was developed to facilitate bidirectional RF communication between the user and the IU. It monitors the recording SW signals in real time and stores them on the computer for offline analysis, simultaneously. Furthermore, the user can program the stimulation parameters through the GUI, including the on- and off-times of the monophasic and biphasic pulses, the amplitudes, and the number of stimulation repetitions. The on-times consist of up to three consecutive pulses with programmable amplitudes and pulse widths, which can be repeated up to 65535 times. Besides, to reach an optimum peak-to-peak amplitude of the SW signals, the user can program and modify the gain of the analog conditioning circuit, wirelessly. The front panel of the GUI for the recording and stimulation functions is shown in Figure 6. Moreover, the user can read the battery voltage of the IU, i.e., the V_BAT_, instantaneously; when it is reduced to a critically low voltage, can switch to the NFC mode for recharging the battery.

## 3. Results

We developed the three units of the system on four-layer printed circuit boards (PCBs). The IU was developed on a folded flex-rigid round PCB with a diameter of 24.5 mm, i.e., the size of a quarter-dollar coin, and supplied it with a lithium-ion coin cell battery with a voltage and capacity of 3.7 V and 85 mAh, respectively. In addition, the CU and the SU were developed on rigid square PCBs with the dimensions of 60 × 60 mm^2^ and 40 × 40 mm^2^, respectively. 

**Figure 7:**
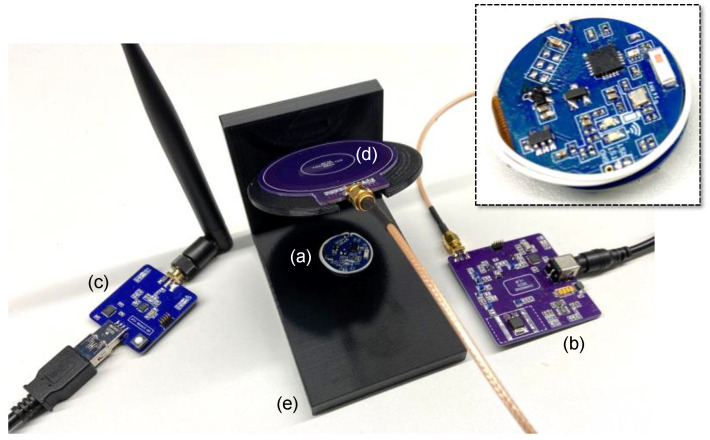
The prototype of the system, consisting of (**a**) a miniature-sized IU with an integrated wire-wound secondary coil in white, (**b**) the CU, (**c**) the SU connected to the computer, (**d**) the PCB-based primary coil connected to the CU, and (**e**) the apparatus to adjust the distance and the misalignment angle between the pair of coils for the adaptive WPT. The inset shows the zoomed IU. In addition, when not considering the SU, it represents the benchtop setup for the validation of the system in the NFC mode of operation.

Regarding the pair of coils on the inductive NFC link, we developed the planar L_1_ on a rigid round PCB, matched it to 50 Ω by a narrow-band capacitive matching network, and integrated it into the CU through a coaxial RF connector. In addition, the L_2_ was developed based on an insulated wire that was wound vertically on the periphery of the disk-shaped IU and integrated into the unit. The parameters of the WPT coils of L_1_ and L_2_ are summarized in Table 2. Additionally, the system prototype, consisting of the IU, the CU, and the SU, along with the inductive NFC coils, is shown in Figure 7.

**Figure 8:**
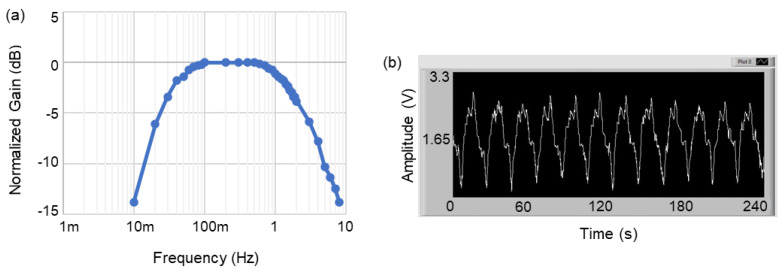
The verification results of the recording module, shown in two steps: (**a**) the normalized frequency response of the analog conditioning circuit on a logarithmic scale, and (**b**) the signal received by the GUI when a sample of SWs previously recorded in vivo was loaded into a function generator and streamed to the recording electrodes of the IU.

We validated the recording function of the system, in two steps. First, we measured the response of the analog conditioning circuit by applying sine waves, with frequencies from 10 mHz to 10 Hz and an amplitude of 250 µV, to the recording electrodes of the IU and measured the output voltage of the circuit, accordingly. The obtained frequency response demonstrated that the IU could successfully condition the SWs within the frequency range of 16 mHz–1.6 Hz. Figure 8a shows the normalized frequency response on a logarithmic scale.

**Figure 9:**
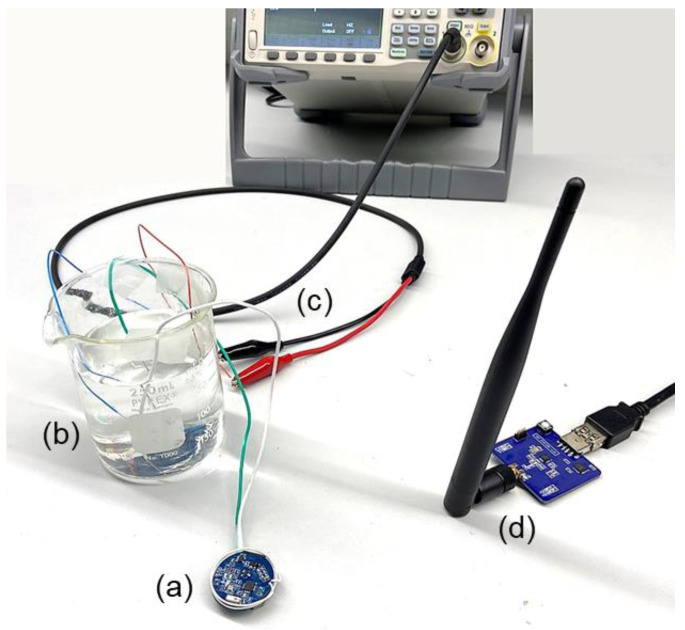
The benchtop setup for the verification of the system in the RF mode of operation is shown: (**a**) the precoating IU, (**b**) the saline solution, (**c**) sample SWs streamed into the solution through a function generator, acquired by the IU, and transmitted to (**d**) the SU.

In the next step, we uploaded SWs data that was previously in vivo to an arbitrary function generator and streamed it to the input electrodes of the analog conditioning circuit through a saline solution [37]. Figure 9 demonstrates the setup for validation of the system in the RF mode of operation. The received signal at the stationary computer was monitored through the GUI and matched the original signal while it was appropriately amplified and filtered. The average power consumption of the IU during recording in the RF mode was measured at equal to 7 mW. The received and monitored SWs in the GUI are shown in Figure 8b.

**Figure 10:**
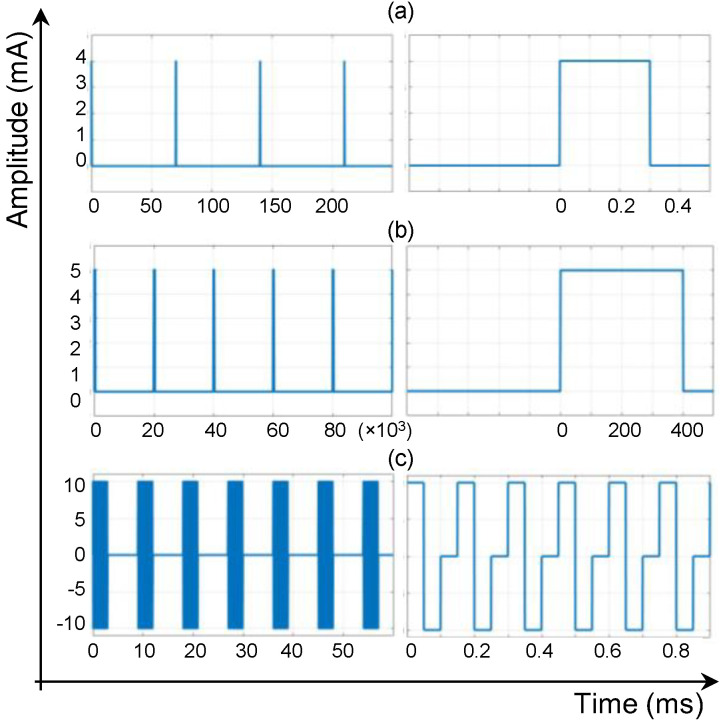
Three different stimulation configurations, generated by the IU, injected into a 1 kΩ resistor, and probed through an oscilloscope. The left- and right-side graphs on each configuration show the stimulations zoomed out and zoomed in over the time axis. (**a**) Monophasic low-energy stimulation with a pulse-width of 300 µs and an amplitude of 4 mA, repeated every 70 ms; (**b**) monophasic high-energy stimulation with a pulse-width of 400 ms, an amplitude of 5 mA, and repeated every 20 s; (**c**) a biphasic pulse train with an on-time of 20 cycles of the sequence of +10 mA, −10 mA, and 0, each with a time-width of 50 μs, and an off-time of 6 ms.

In addition, we verified the capability of the system in generating monophasic stimulation pulses in the range of 0 to 10 mA, along with balanced and unbalanced biphasic current pulses in the range of ± 10 mA, both with minimum pulse widths of 50 µs. We delivered the electrical pulses to a resistor of 1 kΩ and successfully captured the trains of pulses through an oscilloscope. The power consumption of the IU during the stimulation function in the RF mode significantly depends on the stimulation parameters, including the on- and off-times, along with the amplitude, frequency, and pulse-widths of each configuration. Figure 10 shows three configurations of low-energy stimulation, high-energy stimulation, and pulse train recommended for relieving nausea and vomiting, pacing the gut, and modulating the gastric tract as well as the vagus nerve, respectively. Figure 10a illustrates a monophasic low-energy stimulation with a pulse width of 300 µs and an amplitude of 4 mA, which was repeated every 70 ms. In addition, Figure 10b shows a monophasic high-energy stimulation with a pulse width of 400 ms, an amplitude of 5 mA, and a frequency of 50 mHz. Finally, the biphasic pulse train shown in Figure 10c was configured as a sequence of three pulses with amplitudes of +10 mA, −10 mA, and 0, each pulse with a time-width of 50 μs and repeated 20 times. Then, 6 ms was added as the off-time.

**Figure 11:**
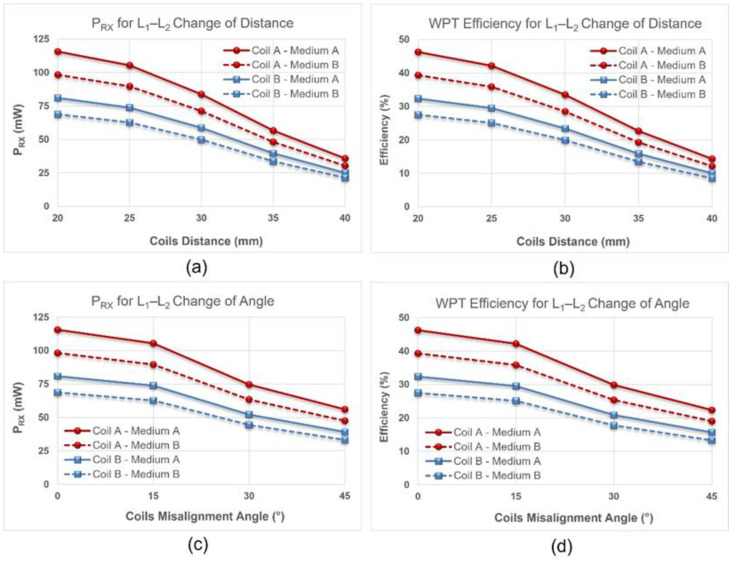
The results of the open-loop WPT measurements are plotted. Coil A and coil B represent the data when L_2_ was 35 mm away from the recording electrodes and when L_2_ was wire-wound on the periphery of the IU, respectively. Medium A and Medium B represent the data when the inductive link mediums were air and chicken breast, respectively. For a constant transmitted power of 250 mW, the plots show (**a**) the IU’s P_RX_ when the distance between the L_1_ and L_2_ coils was increased from 20 mm to 40 mm, in steps of 5 mm, (**b**) the WPT efficiency for the same L_1_–L_2_ distancing, (**c**) the P_RX_ when the L_1_–L_2_ horizontal angle increased from 0° to 45° in steps of 15° at a fixed distance of 20 mm, and (**d**) the WPT efficiency for the same L_1_–L_2_ change of horizontal angle.

Furthermore, we validated the wireless power and data transfer function of the system in the NFC mode, in two steps. First, we verified the performance of open-loop WPT, i.e., without instantaneous adjustment of the CU’s output power. To this end, we programmed the resistance of the digital potentiometer at the CU, such that the RF power amplifier generated a constant power of 24 dBm (250 mW). Then, we measured the received power (P_RX_) by the IU and calculated the efficiency of the WPT link, while we increased the distance between L_1_ and L_2_ from 20 mm to 40 mm in steps of 5 mm, and increased the horizontal angle of L_1_ from 0° to 45° in steps of 15° at a fixed distance of 20 mm. Regarding the significance of eddy current losses, we verified the open-loop WPT for two configurations, when L_2_ was wire-wound on the periphery of the IU and when it was spaced from the recording electrodes by 35 mm, and compared the results. In addition, to mimic the condition of in vivo studies with the presence of live tissue between the pair of coils, we used chicken breast along with air for the mediums on the WPT link. In the scenario where L_2_ was spaced from the IU, we increased the L_1_–L_2_ distance from 20 mm to 40 mm, and the IU’s P_RX_ decreased from 115 mW to 35 mW and from 98 mW to 30 mW for the mediums of air and chicken breast, respectively. For the same configuration of L_2_, P_RX_ decreased from 115 mW to 55 mW and from 98 mW to 47 mW when we increased the L_1_ horizontal angle from 0° to 45°, for the mediums of air and chicken breast, respectively. Furthermore, in the scenario where L_2_ was wire-wound vertically on the periphery of the IU, the P_RX_ decreased from 80 mW to 25 mW and from 68 mW to 21 mW for the respective mediums of air and chicken breast, when we changed the L_1_ – L_2_ distance from 20 mm to 40 mm. For the same, recent, configuration, P_RX_ decreased from 80 mW to 39 mW and from 68 mW to 33 mW, while we changed the L_1′_s horizontal angle from 0° to 45°. The data plotted in Figure 11 summarizes the results of the open-loop WPT measurements. 

**Figure 12:**
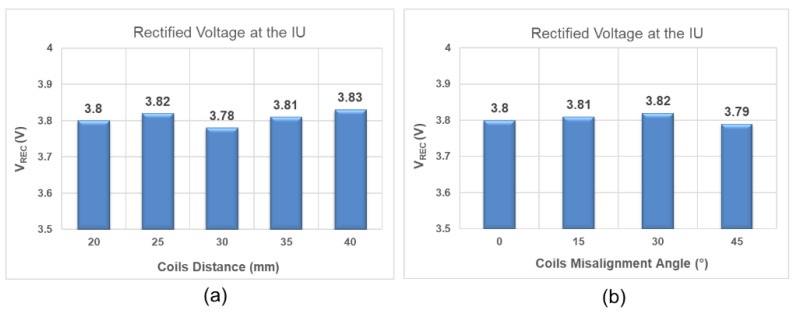
The results of the adaptive closed-loop WPT measurements in the NFC mode were plotted. The plots show (**a**) the V_REC_ at the IU when the distance between the L_1_ and L_2_ coils was increased from 20 mm to 40 mm in steps of 5 mm; (**b**) the V_REC_ at the IU when at a fixed distance of 20 mm; the L_1_–L_2_ horizontal angle increased from 0° to 45° in steps of 15°.

In the second step, we validated the adaptive closed-loop wireless power and data transfer. Between the two configurations for the IU’s coil, i.e., L_2_ spaced from the IU for 35 mm and L_2_ alone was wire-wound on the periphery of the device; we chose the latter with a worse WPT efficiency with the chicken breast for the medium on the WPT link. First, we increased the L_1_–L_2_ distance from 20 mm to 40 mm, in steps of 5 mm, and measured the V_REC_ at the IU for each step. Based on the 5 measurements, we calculated a mean value of 3.808 V and a percentage error of 0.21%. Furthermore, we measured the V_REC_ when the L_1_L_2_ horizontal angle was increased from 0° to 45° in steps of 15° at a fixed distance of 20 mm. The mean value of 3.805 V and a percent error of 0.13% were what we calculated based on the misalignment measurements. Figure 12 summarizes the results of the adaptive closed-loop WPT measurements in the NFC mode. In an additional experiment, we monitored the temperature rise of the chicken breast as the medium during the closed-loop WPT validation. For the time in which the CU recharged the IU’s battery and increased its voltage from 3.3 V to 4.2 V in approximately 45 min, the elevation of temperature in the chicken breast was limited to 1 °C in reference to the room temperature.

## 4. Discussion

To comprehensively understand the pathophysiological significance of functional gastric diseases, digestive disorders, and dysmotility, chronic studies on conscious subjects in both the fasting and fed states are essential. In addition, due to the technological limitations of current systems, the feasibility of a detailed study on the therapeutic potentials of electroceutical methods and electrical pulses remains low. To achieve these goals, the development of IMDs with a reliable source of power to conduct long-term research on the electrophysiology of the gastric system is substantially required. To this end, a WPT link guarantees the continued operation of medical implants and eliminates removing the IMD when its battery is dead. The range-to-size ratio of medical implants determines the type of WPT technology applicable to that category of IMDs, i.e., resonant inductive and ultrasound IMDs [38,39,40,41,42]. Based on this ratio for gastric IMDs, resonant inductive links provide the optimum WPT efficiency [20]. However, adverse health effects and safety concerns due to the exposure of live tissues to the EM waves in the inductive WPT need to be addressed and resolved appropriately.

We developed a miniature wireless implantable system for simultaneously recording and stimulating the gastric SWs in freely behaving rodents. On average, the number of cycles of the SWs per minute in humans and in rodents is 3 and up to 5, which cycles are translated to 50 mHz and 83 mHz, respectively. The frequency response of the analog conditioning circuit indicates that the system can acquire SWs at slower and/or faster paces; therefore, they can also detect and monitor the gastric disorders of bradygastria and tachygastria [43]. In addition, the system can generate stimulation pulses with various amplitudes and pulse-widths of up to ± 10 mA and as narrow as 50 µs, respectively, applied for modulating the gastric tract, as well as the vagus nerve, and for relieving the various symptoms of gastroparesis and motility disorders. 

Moreover, the system features an RFID-based closed-loop NFC to implement an adaptive WPT, i.e., in the presence of body movements and stomach motility, it delivers constant power to the IU, while in reference to a V_REF_, it prevents a decreased or excessive amount of power being received by the IU. In addition, the system complies with the safety limits of the SAR discussed in the IEEE standard and retains a steady-state temperature elevation of less than 1 °C. Depending on the duration and the magnitude of power density beyond the SAR, long-term exposure of live subjects to these EM fields results in an elevation in the temperature in the medium and can cause adverse health effects and irreversible thermal damage to the tissue and skin [21].

In addition to the sensitivity of the resonant inductive WPT to the change of distance and misalignment between the pair of coils, eddy currents are a major source of losses and are the cause of a significant decrease in the efficiency of the WPT link. A comparison between the WPT efficiency for the configurations of an L_2_ spaced from the IU and an L_2_ wire-wound on the periphery of the IU is shown in Figure 11, It was observed that the efficiency is reduced by approximately 15–20% for either of the mediums. This efficiency drop is effectively caused by the interference in the circuitry and the copper layers of the PCB of the IU with the EM field in the NFC mode. 

Furthermore, the firmware of the IU was developed in such a way that it operates in either the RF mode or the NFC mode. In other words, the system pauses the recording and stimulation functions during wireless charging. Regarding the close proximity of L_2_ and the recording electrodes, when L_2_ is wire-wound on the periphery of the IU, concurrent running of the two modes may affect the signal-to-noise ratio of the SW acquisition. Otherwise, the system is capable of running both RF and NFC modes simultaneously, without any conflict in terms of functions. The concurrent operation of the RF and NFC modes can be utilized in live subjects with possible spacing between the IU’s coil and the recording electrodes. 

In the next research steps, we will conduct long-term in vivo studies on the neurophysiology of the GI tract, the diagnosis of functional gastrointestinal disorders, and the efficacy of electroceutical therapies. In the context of system implantation in rodents, we will use cardiac pacing wires for the electrodes of the recording and stimulation circuits. Both pairs of electrodes will be sutured onto the serosa of the GI tract and directly fixed by a surgical knot [44]. 

## Figures and Tables

**Table 1:** Incremental/decremental steps for the tap number of the digital potentiometer at the CU.

Last Tap Number	Increment Steps (V_REC_ > V_REF_)	Decrement Steps (V_REC_ < V_REF_)
0 ≤ N_LAST_ * < 24	2	2 (or jump to tap 0 if N_LAST_ − 2 < 0)
25 ≤ N_LAST_ < 88	3	3
89 ≤ N_LAST_ < 158	7	7
159 ≤ N_LAST_ < 255	10 (or jump to tap 255 if N_LAST_ + 10 > 255)	10

* The last wiper position of the digital potentiometer stored in the CU’s microcontroller.

**Table 2:** Specifications of the inductive NFC coils.

Parameters	L_1_	L_2_
Inductance (µH)	3.43	0.189
Quality Factor	106	32.2
Outer Diameter (mm)	60	25
Inner Diameter (mm)	49	-
Width (mm)/Thickness (µm)	0.5/35	-
Diameter (mm)	-	0.5
Number of Turns	5	2
Type of Coil	PCB	Wire-wound

## Data Availability

The data presented in this study are available on request from the corresponding author.
